# Target c-Myc to treat pancreatic cancer

**DOI:** 10.1080/15384047.2021.2017223

**Published:** 2022-01-03

**Authors:** Moein Ala

**Affiliations:** School of Medicine, Tehran University of Medical Sciences (TUMS), Tehran, Iran

**Keywords:** C-myc, pancreatic cancer, BET inhibitor, chemoresistance, KRAS

## Abstract

C-Myc overexpression is a common finding in pancreatic cancer and predicts the aggressive behavior of cancer cells. It binds to the promoter of different genes, thereby regulating their transcription. C-Myc is downstream of KRAS and interacts with several oncogenic and proliferative pathways in pancreatic cancer. C-Myc enhances aerobic glycolysis in cancer cells and regulates glutamate biosynthesis from glutamine. It provides enough energy for cancer cells’ metabolism and sufficient substrate for the synthesis of organic molecules. C-Myc overexpression is associated with chemoresistance, intra-tumor angiogenesis, epithelial-mesenchymal transition (EMT), and metastasis in pancreatic cancer. Despite its title, c-Myc is not “undruggable” and recent studies unveiled that it can be targeted, directly or indirectly. Small molecules that accelerate c-Myc ubiquitination and degradation have been effective in preclinical studies. Small molecules that hinder c-Myc-MAX heterodimerization or c-Myc/MAX/DNA complex formation can functionally inhibit c-Myc. In addition, c-Myc can be targeted through transcriptional, post-transcriptional, and translational modifications.

## Introduction

Pancreatic cancer constitutes 1.8% of all cancers and is responsible for 4.6% of cancer-related death. Its incidence, prevalence, and mortality markedly increased during recent years.^1^ Pancreatic ductal adenocarcinoma is the most common form of pancreatic cancer and accounts for 90% of all exocrine pancreatic cancers.^2^ Pancreatic ductal adenocarcinoma has a low 5-year survival rate ranging from 4.2% to 17.4% among patients of all stages.^3^ Despite great advances in chemotherapeutic methods, yet non-surgical treatments remain less effective for pancreatic ductal adenocarcinoma.^3^ There are several types of pancreatic cancer such as pancreatic ductal carcinoma, mucinous tumors, and pancreatic neuroendocrine tumors. Pancreatic cancer in this review is considered pancreatic ductal adenocarcinoma unless otherwise mentioned.

According to the latest studies, six months of gemcitabine and capecitabine or FOLFIRINOX regimen consisting of folinic acid, fluorouracil, irinotecan, and oxaliplatin are the first-line adjuvant chemotherapeutic management for pancreatic cancer. Likewise, nab-paclitaxel can be used for higher stages of this cancer. Despite many efforts, the chemotherapeutic response for pancreatic cancer remains weak and limited.^4–7^ Regarding the importance of pancreatic cancer and limited achievements in its treatment, new chemotherapeutic agents are needed to improve its management. Herein, in this review, a new target for chemotherapy with pronounced involvement in pancreatic cancer is discussed.

C-Myc oncoprotein has been implicated in the pathogenesis of several cancers such as pancreatic cancer, hepatocellular carcinoma, and breast cancer.^8–10^ Stabilized c-Myc can reprogram key genes expression involved in the metabolism, mitochondrial and ribosomal biogenesis, differentiation, apoptosis, and proliferation of cancer cells.^8,11^ C-Myc is located on human chromosome 8 and regulates genes expression by binding to their enhancer box (E-box).^12^ C-Myc is downstream of KRAS and its overexpression is found in 43.5% of primary pancreatic cancers.^13,14^ KRAS proto-oncogene is frequently found in tumor samples of patients with pancreatic cancer with more prevalence among patients with higher stages of the tumor. Even, the presence of KRAS mutation prognosticates shorter disease-free survival in patients with localized tumor.^14^ Recently, it was observed that activation of c-Myc is enough to convert indolent pancreatic intraepithelial neoplasm into pancreatic cancer in mice.^15^ Suppression of c-Myc in resistant pancreatic cancer could improve response to ordinary chemotherapeutic agents in preclinical studies.^10^

In this review, it is discussed how c-Myc is involved in the biology and progression of pancreatic cancer. Furthermore, this review attempts to illuminate the interaction between c-Myc and other molecules in pancreatic cancer. Finally, possible mechanisms to target c-Myc are briefly discussed.

## The effect of c-Myc on cancer cells metabolism

C-Myc helps tumor cells to increase their glucose uptake and accelerate their lactate production instead of using the Krebs cycle for energy metabolism, even in the presence of a sufficient amount of oxygen. This effect is known as the Warburg effect or aerobic glycolysis.^16^ Glycolysis can provide a large amount of lactate that can be used for the anabolic processes of cancer cells.^17^ C-Myc can enhance pyruvate kinase isoenzyme type M2 (PKM2) expression to improve tumor cell glycolysis.^18^ Further, c-Myc-induced overexpression of PKM2 was shown to augment tumor cells proliferation, invasion, and chemoresistance.^19^ Similarly, it was shown that c-Myc, through repression of a lncRNA, IDH1-AS1, can downregulate isocitrate dehydrogenase 1 (IDH1). Increased activity of IDH1 increases α-ketoglutarate and downregulates HIF-1α. Hence, c-Myc through inhibition of IDH1 can upregulate HIF-1α and lactate production.^20^ Consistently, it was shown that c-Myc downregulation leads to inhibition of HIF-1α signaling in pancreatic cancer.^21^ In response to hypoxia, HIF-1α provokes glycolysis in pancreatic cancer cells to provide enough ATP and lactate.^17^ The collaboration between c-Myc and HIF-1α is responsible for the Warburg effect and lactate production in tumor cells.^20^ C-Myc also promotes the expression of lactate dehydrogenase A (LDHA) to convert pyruvate to lactate at the end of glycolysis.^22^ Inhibition of c-Myc downregulates LDHA expression in cancer cells.^23^ LDHA and PKM2 overexpression is a common finding in pancreatic cancer and enhances cancer cells proliferation and poor outcome.^24,25^ Consistently, treatment with either LDHA or PMK2 inhibitor could improve the efficacy of gemcitabine in pancreatic cancer, promote the apoptosis of pancreatic cancer cells and suppress their invasive behavior.^26–28^

C-Myc downregulates the expression of thioredoxin-interacting protein (TXNIP), which is a tumor suppressor and a potent inhibitor of glucose consumption.^29^ Repression of TXNIP expression strengthens the proliferative and metastatic capacity of pancreatic cancer.^30^ The inhibitory effect of TXNIP on glycolysis weakens the metastatic capacity of tumor cells.^31^ In addition, c-Myc promotes the expression of glucose transporters (GLUTs) such as GLUT1.^9,32^ GLUT1 overexpression predicts worse 3-year and 5-year overall survival and disease-free survival of solid tumors.^33^ By enhancing glucose uptake during glucose starvation, it can switch cellular metabolism from β-oxidation to glycolysis. Even, this property was shown to be protective against myocardial ischemia/reperfusion in an animal study. Moreover, it was observed that c-Myc can downregulate PGC-1α which is the major regulator of mitochondria biogenesis and negatively regulates glycolysis and Warburg effect.^34–37^ Consistently, it was shown that higher KRAS and c-Myc expression are associated with increased glucose uptake among patients with pancreatic cancer. Indeed, KRAS negatively regulates F-box/WD repeat-containing protein 7 (FBW7), a tumor suppressor gene. FBW7 also negatively regulates c-Myc in pancreatic cancer. Upregulation of KRAS abrogates the inhibitory effect of FBW7 on c-Myc and increases glucose uptake.^38^ Increased expression of GLUTs in cancer cells lets them uptake a higher amount of glucose in their microenvironment. This characteristic improves their survival during glucose starvation and provides enough energy for their rapidly progressive cell cycle. C-Myc is a master regulator of tumor cells metabolism which increases glucose consumption in the tumor microenvironment to provide enough energy and produce a sufficient amount of lipids, proteins, and nucleic acids for tumor cells proliferation.^39^ Interestingly, Sato *et al*. uncovered that hyperglycemia upregulates c-Myc expression in pancreatic cancer cells and expedites pancreatic cancer growth in mice.^40^

**Figure 1.**
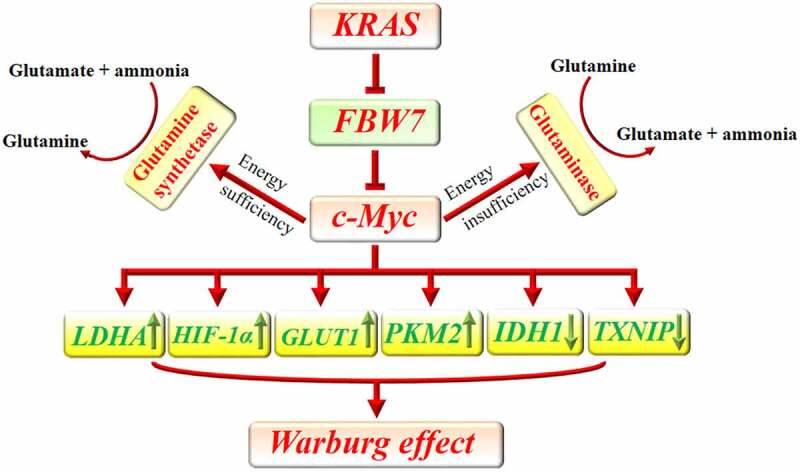
Implication of c-Myc in metabolic reprogramming of pancreatic cancer. KRAS downregulates FBW7 to prevent its inhibitory effect on c-Myc. C-Myc can increase the uptake of glucose by increasing the expression of GLUT1 and decreasing the expression of TXNIP. Furthermore, c-Myc promotes the expression of LDHA and PKM2 and decreases IDH1 to enhance glycolysis. Additionally, c-Myc potentiates HIF-1α which can similarly accelerate glycolysis. In the presence of energy insufficiency, c-Myc can promote glutaminase expression which subsequently increases glutamate bioavailability as a source of energy. In the presence of sufficient source of energy, c-Myc promotes the expression of glutamine synthetase and increases the available amount of glutamine which can be used for anabolic purposes in cancer cells.

Gao *et al*. uncovered that c-Myc represses miR-23a and miR-23b to increase the expression of mitochondrial glutaminase in human cancer cells. The enzyme converts glutamine to glutamate which will be finally used for ATP production through the tricarboxylic acid (TCA) cycle. This function of c-Myc provides energy for cancer cells during energy depletion and protects them against oxidative stress.^41^ Cancer cells rely on glutamine for their anabolic process. Enough amount of glutamine guarantees cancer cells survival and proliferation. In addition, glutamine is critically involved in the uptake of essential amino acids and sustained activation of mTOR.^42^ Interestingly, it was uncovered that c-Myc also promotes the gene transcription of glutamine synthetase via demethylation of its promoter. In the presence of a sufficient amount of glucose in tumor cells’ microenvironment, glutamine synthetase increases de novo production of glutamine from glutamate and ammonia. Increased expression of glutamine synthetase reverses glutamine deficiency, improves tumor cells viability, and enhances nucleotide and amino acid transport.^43^ Similarly, it was observed that higher blood glutamine uptake by pancreatic cancer cells is associated with their invasive behavior and progression from pancreatic intraepithelial lesions to invasive pancreatic cancer.^44^ C-Myc reprograms metabolic pathways of pancreatic cancer cells to overcome their limitations (Figure 1).

### C-Myc contributes to tumor angiogenesis, metastasis, and chemoresistance

Angiogenesis is an indispensable necessity for tumor growth, invasion, and metastasis. Normally, tumor cells enhance the expression of angiogenic factors such as VEGF to improve angiogenesis. The increased vascular network provides more blood supply for tumor cells proliferation and lets them migrate to other organs.^45^ C-Myc protects against endothelial dysfunction and enhances angiogenesis.^46^ It is also critically involved in tumor angiogenesis. C-Myc deletion is associated with decreased vascularization within the tumor. It can positively regulate the expression of VEGF and other molecules involved in angiogenesis.^47,48^ However, Dews *et al*. claimed that the effect of c-Myc on angiogenesis is not dependent on VEGF. Instead, it mainly downregulates the expression of anti-angiogenic factors such as thrombospondin-1 (Tsp1) and connective tissue growth factor (CTGF). C-Myc increases the expression of miR-17-92 and subsequently prevents the expression of Tsp1 and CTGF.^49^ Interestingly, Chen *et al*. revealed that c-Myc can post-transcriptionally promote the expression of HIF-1α and potentiate HIF-1α/VEGF axis to improve angiogenesis.^50^ In response to hypoxia, HIF-1α stimulates VEGF expression and accelerates angiogenesis.^51^ Targeting c-Myc as a positive regulator of angiogenesis can impair tumor cells’ metabolism and decrease their metastatic potential.

It was observed that fibroblast growth factor (FGF) binding protein 1 (FGFBP1) plays a pivotal role in the proliferation and metastasis of pancreatic cancer cells and negatively correlates with the survival of patients with pancreatic cancer. It was shown that FBW7 through a c-Myc-dependent manner downregulates FGFBP1. Also, downregulation of FBW7 results in c-Myc/FGFBP1 axis overactivity.^52^ FGFBP1 can contribute to angiogenesis which facilitates cancer cells migration.^53^

C-Myc enhances the expression of several transcription factors such as zinc finger E-box binding homeobox (ZEB) 1, ZEB2, Snail1, Snail2, and Twist to accelerate EMT.^54^ These downstream transcription factors can promote the transcription of EMT-associated genes such as MMPs, N-cadherin, and fibronectin. They also repress the transcription of E-cadherin, plakophilin, occludin, and cytokeratin to accelerate EMT of cancer cells.^55^ Consistently, it was shown that c-Myc increases matrix metallopeptidase (MMP) expression and decreases E-cadherin expression to augment EMT of pancreatic cancer cells.^56–58^ Increased expression of MMP and decreased expression of cell adhesion molecules can enhance cancer cells’ mobility.^59^ Therefore, c-Myc can enhance angiogenesis and accelerate EMT to facilitate cancer cells metastasis.

Pancreatic cancer poorly responds to chemotherapy and chemoresistance is the main obstacle for pancreatic cancer chemotherapy.^59,60^ Alteration of ^1^metabolic profile of cancer cells and activation of compensatory pathways seems to be involved in such behavior. C-Myc overexpression can strongly enhance pancreatic cancer cells viability against chemotherapy agents.^10,61^ Meanwhile, c-Myc downregulation or inhibition can improve chemoresistance and increase the efficacy of several chemotherapeutic agents such as nab-paclitaxel and gemcitabine.^61–63^ Therefore, simultaneous inhibition of c-Myc contributes to overcoming pancreatic cancer chemoresistance.

### The correlation between c-Myc and oncogenic signaling pathways in pancreatic cancer

Aberrant activation of different signaling pathways is involved in the pathogenesis of pancreatic cancer.^64^ Additionally, inhibition of these proliferative signals showed satisfactory efficacy in preclinical studies of pancreatic cancer.^64^ As mentioned previously, KRAS mutation is a major propellant for the initiation and progression of pancreatic cancer. Mutant KRAS activates several signaling pathways in the pancreas to reprogram pancreatic acini into pancreatic cancer.^65^ Transgenic overexpression of KRAS resulted in the development of invasive pancreatic cancer in mice. Ablation or inhibition of c-Myc led to marked tumor shrinkage in these KRAS-mutated mice and increased cancer cells apoptosis.^66^ Likewise, downregulation of c-Myc inhibited KRAS-mediated pancreatic cancer cells growth in cell culture.^67^

KRAS activates phosphatidylinositol-3-kinase (PI3K) and WNT signaling pathways in pancreatic cancer.^68–70^ PI3K/protein kinase B (AKT)/mammalian target of rapamycin (mTOR) signaling pathway is a major oncogenic pathway in pancreatic cancer which promotes tumor cells proliferation, epithelial-mesenchymal transition, and angiogenesis.^71^ Interestingly, it was shown that pan PI3K inhibitor or combinatorial PI3K/mTOR inhibitor can significantly decrease c-Myc expression in pancreatic cancer and inhibit cancer progression.^71,72^ In addition, c-Myc inhibition could significantly abrogate PI3K/AKT/mTOR-mediated proliferative response in pancreatic cancer.^73^ It was shown that protein phosphatase 2A (PP2A), a tumor suppressor gene, can downregulate c-Myc expression in pancreatic cancer and improve pancreatic cancer response to mTOR inhibitor. Meanwhile, it was shown that c-Myc overexpression can increase therapeutic resistance to mTOR inhibition. Further, decreased c-Myc expression improves cancer cells’ response to mTOR inhibition.^74,75^ C-Myc enhances mTOR-mediated 4EBP1 phosphorylation in the first stages of tumorigenesis.^76^ C-Myc blocks the inhibitory effect of tuberous sclerosis complex 2 (TSC2) on mTOR.^77^ In exchange, mTOR/S6/eIF4B regulates the translation of c-Myc in pancreatic cancer.^78^ The collaborative function of c-Myc and mTOR increases vascular endothelial growth factor (VEGF) expression in pancreatic neuroendocrine tumor which enhances intra-tumor vascular network and has been associated with lymph node metastasis.^79^ Furthermore, low expression of phosphatase and tensin homologue (PTEN) and liver kinase B1 (LKB1) in pancreatic neuroendocrine tumors resulted in decreased sensitivity to mTOR inhibitors and increased expression of c-Myc. LKB1 can inhibit mTOR/c-Myc through activation of AMPK/TSC2 signaling.^80^ Since AMPK negatively regulates mTOR and mTOR positively regulates c-Myc, AMPK activation and c-Myc inhibition both abrogated resistance to mTOR inhibitor.^80^ PTEN inhibits AKT/mTOR signaling to prevent cancer cells proliferation.^80^ It was shown that PTEN downregulates AKT/mTOR signaling pathway to decrease VEGF expression, intra-tumor angiogenesis, and tumor cells migration in pancreatic cancer.^81^ Mutual interaction between c-Myc and mTOR can promote ribosomal protein synthesis in tumor cells and provide their metabolic need for proliferation.^82^

Dysregulation of WNT/β-catenin signaling pathway leads to proliferative response, invasion, metastasis, and chemoresistance in pancreatic cancer.^83,84^ KRAS/WNT/β-catenin pathway increases c-Myc expression in pancreatic cancer to promote cancer cell proliferation.^85,86^ C-Myc overexpression provokes ribosomal biogenesis and c-Myc inhibition can partly reverse the effect of WNT/β-catenin pathway in pancreatic cancer.^85^ WNT/β-catenin signaling pathway relies on c-Myc for parts of its oncogenic function and inhibition of this signaling pathway suppresses the expression of c-Myc in pancreatic cancer.^87–89^ Inhibition of WNT/β-catenin signaling pathway seems to be an effective method to prevent the oncogenic effects of c-Myc in pancreatic cancer.^90^ It was shown that c-Myc can increase the transcription of WNT-related genes such as lymphoid enhancer-binding factor 1(LEF1) and augment WNT/β-catenin signaling pathway in cancer cells.^91,92^

**Figure 2.**
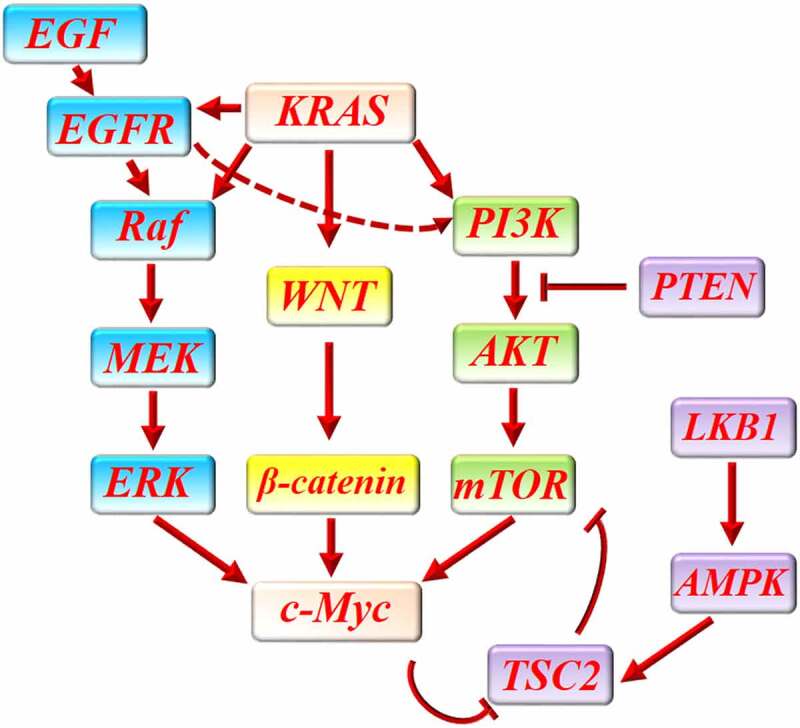
The interaction between c-Myc and main proliferative signaling pathways in pancreatic cancer. KRAS activates several signaling pathways in pancreatic cancer to promote cancer cells proliferation. WNT/β-catenin pathway, PI3K/AKT/mTOR pathway and Raf/MEK/ERK are three major proliferative pathways which are activated by KRAS in pancreatic cancer. In addition, KRAS enhances EGF/EGF receptor-mediated activation of PI3K/AKT/mTOR and Raf/MEK/ERK pathway. All of these pathways can finally increase the expression of c-Myc to promote cancer cells proliferation. LKB1/AMPK/TSC2 and PTEN can protect against pancreatic cancer through downregulation of PI3KAKT/mTOR pathway. C-Myc can increase mTOR function by abrogating the inhibitory effect of LKB1/AMPK/TSC2 on mTOR.

Extracellular signal-regulated kinases (ERK) inhibition leads to growth arrest in KRAS-mutant pancreatic cancer which is partly mediated through c-Myc degradation.^93^ Additionally, it was shown that lncRNAs can modulate ERK function to regulate c-Myc and pancreatic cancer cells proliferation. For instance, LINC00261 inhibits ERK-mediated c-Myc expression and suppresses pancreatic cancer cells proliferation.^94^ Inhibition of ERK and attenuation of ERK-mediated c-Myc overexpression improved gemcitabine-induced cytotoxicity in pancreatic cancer.^62^1.Figure 1 and Figure 2 are misplaced. Figure 1 must be marked as Figure 2 and Figure 2 must be inserted as figure 1. The caption for Figure 1 (implication of c-Myc in metabolic reprogramming ....) is correctly marked as Figure 1 but its associated figure is misplaced. Also, the caption for Figure 2 is correct but its associated Figure is wrongly marked as Figure 1. In summary, Figures but not their captions must be reordered. Their current cations do not belong to them.

As mentioned previously, KRAS mutation is a major propellant for the initiation and progression of pancreatic cancer. Mutant KRAS activates several signaling pathways in the pancreas to reprogram pancreatic acini into pancreatic cancer.^65^ In addition to PI3K/AKT/mTOR and WNT/β-catenin, Mutant KRAS activates Raf/MEK/ERK signaling pathway to upregulate c-Myc.^93,95^ ERK was shown to be involved in the epidermal growth factor (EGF)/EGF receptor-mediated increase in c-Myc expression which has been associated with development, growth, and invasion of pancreatic cancer.^96,97^ EGF receptor also activates PI3K pathway to stimulate cancer cells proliferation.^98^ Interestingly, it has been elucidated that KRAS promotes EGFR signaling to increase c-Myc signaling in pancreatic cancer. However, EGFR signaling inhibition was not sufficient to inhibit EKR and PI3K signaling after KRAS activation.^99^

Inhibition of each one of these oncogenic pathways in pancreatic cancer can provide a limited benefit or is associated with chemoresistance through upregulation of other pathways, while, their simultaneous inhibition, for example, concurrent inhibition of PI3K and MEK pathways can conceivably improve treatment efficacy.^93,100^ Targeting c-Myc as the common downstream of several oncogenic pathways in pancreatic cancer may bring more favorable outcomes than targeting each one of them, alone (Figure 2).

### C-Myc downregulation makes pancreatic cancer cells susceptible to oxidative stress

Uncontrolled release of reactive oxygen species (ROS) is immensely involved in the anti-cancer effect of radiotherapy and chemotherapeutic agents.^101^ An excessive amount of ROS damages cancer cells organelles and biomacromolecules and endangers their DNA integrity leading to cancer cells death.^101^ Likewise, it has been found that cancer cells particularly pancreatic cancer cells recruit specific mechanisms to augment their defense mechanisms against oxidative stress.^102^ This capability has been implicated in the chemoresistance of pancreatic cancer cells.^102^ In addition, it was found that attenuation of these self-defense mechanisms can vigorously abrogate the chemoresistance of pancreatic cancer cells to routine chemotherapeutic agents such as 5-fluorouracil and gemcitabine.^102^ Regarding the effect of c-Myc on aerobic glycolysis and mitochondrial respiration, c-Myc upregulation is a major defense mechanism against oxidative stress in pancreatic cancer.^103^ Impaired mitochondrial oxidative phosphorylation results in oxidative burst and leads to cancer cells death.^104^ It was found that c-Myc downregulation contributes to mitochondrial respiration, accelerates oxidative stress, and contributes to overcoming chemoresistance.^105^ Furthermore, c-Myc binds to the promoter site of nuclear factor erythroid 2–related factor 2 (Nrf2) and enhances its expression.^106^ Nrf2 is the main transcription factor for numerous endogenous antioxidants.^106^ The interaction between c-Myc and Nrf-2 was shown to be crucially involved in the survival of pancreatic cancer cells.^106^ These findings show that c-Myc overexpression not only expedites tumor cells proliferation but also protects them against stress conditions.

### C-Myc interacts with cyclin-dependent kinase (CDK) to enhance pancreatic cancer cells proliferation

CDKs are serine/threonine kinases that interact with cyclins to regulate cell cycle progression. Dysregulation and overactivity of CKDs are characterized by excessive proliferation and are a hallmark of cancers.^107^ Interestingly, it was uncovered that tumor cells use c-Myc overexpression as a resistance mechanism against CDK inhibitors. For instance, CDK4/6 inhibition has been associated with increased mTOR signaling and led to overexpression of c-Myc. These alterations reprogrammed cancer cells’ metabolism and improved glycolysis.^108^ In return, c-Myc can potentiate the cyclin-CDK system in pancreatic cancer cells by increasing the expression of its members such as cyclin D1, CDK1, and cyclin B1, and decreasing the expression of P21, cyclin-dependent kinase inhibitor 1.^109^ Besides, it has been shown that some CDKs such as CDK9 are involved in the expression of c-Myc and their inhibition will result in decreased c-Myc abundance in pancreatic cancer.^67^ These CKDs possess a pivotal role in the transcription initiation and elongation of c-Myc mRNA.^110,111^ Therefore, c-Myc can interact with the cyclin-CDK system to accelerate cell cycle progression and induce chemoresistance.

### Targeting c-Myc improves immunotherapy in pancreatic cancer

Immune checkpoint blockade has shown acceptable efficacy in different cancers but not in pancreatic cancer.^112^ The presence of a markedly immunosuppressive and immune-privileged microenvironment and the absence of an effective level of T cells infiltration in pancreatic cancer have been implicated in the weak response of pancreatic cancer to immune checkpoint blockade.^112^ Hence, finding new solutions to overcome the immune-evasion characteristic of pancreatic cancer can greatly help its treatment.^112^

Previously, it has been found that c-Myc contributes to cancer cells’ immune evasion. C-Myc overexpression prevents immune cells from attacking tumor cells.^113^ Interestingly, the expression of c-Myc and programmed death ligand 1 (PD-L1) was positively correlated in the tissue samples obtained from patients with esophageal cancer.^113^ Furthermore, c-Myc overexpression, silencing, and inhibition replicated the same pattern on the expression of PD-L1 in cell culture of esophageal cancer, suggesting that c-Myc is a major regulator of PD-L1.^113^ Likewise, downregulation of c-Myc suppressed PD-L1 expression in non-small cell lung carcinoma and attenuated tumor cells immune-evasion.^114^ PD-L1 is vigorously expressed by tumor cells and acts as a defense mechanism.^115^ It binds to programmed death 1 (PD-1) on immune cells particularly on T cells surface and instigates their death.^115^ Inhibition of c-Myc enhanced the abundance of CD4+ cells, CD8+ cells, B cells, dendritic cells, and natural killer cells in the tumor microenvironment, in a mice model of prostate cancer.^116^ Furthermore, c-Myc inhibitor potentiated the anti-cancer efficacy of anti-PD-1 immunotherapy.^116^ Similarly, it was found that c-Myc expression and PD-L1 expression are positively correlated in the tissue specimens obtained from 87 patients with pancreatic cancer.^117^ In addition, activation of c-Myc in the mice model of pancreatic intraepithelial neoplasm has been associated with decreased abundance of infiltrating T cells and higher incidence of pancreatic cancer.^15^ Consistently, inhibition of c-Myc in mice or silencing it in cell culture downregulated the expression of PD-L1 in pancreatic cancer.^117^ Moreover, inhibition of c-Myc and PD-L1 blockade exerted a synergistic effect on the mice model of pancreatic cancer.^117^ C-Myc is a major transcription factor for PD-L1 in pancreatic cancer and c-Myc knockdown profoundly decreases PD-L1 expression in pancreatic cancer.^118^

C-Myc also increases CD47 expression in pancreatic cancer.^119^ C-Myc increases the transcription of CD47 in cancer cells. CD47 is located on the surface of cancer cells and interacts with signal-regulatory protein alpha (SIRP-α) to induce tolerance in immune cells, prevent cancer cells phagocytosis, and prolong cancer cells survival in pancreatic cancer.^120,121^ CD47 blockade enhanced the antitumor function of CD8 + T cells and macrophages in the mice model of pancreatic cancer.^122^ Likewise, immunotherapy with CD47-CAR-T cells significantly reduced pancreatic cancer cells population and prevent pancreatic cancer growth in mice.^123^

Interestingly, c-Myc is involved in the homeostasis and competent function of T regulatory cell which acts as the main player in tumor cells immune-evasion.^124,125^ Downregulation of c-Myc can impair the immunosuppressive function of T regulatory cells, thereby enhancing the anti-cancer capacity of effector T cells.^124^ These findings reveal that c-Myc is widely involved in the immune cells-tumor cells cross-talk and downregulation of c-Myc can enormously improve immunotherapy in pancreatic cancer.

### Interaction between c-Myc and long non-coding RNAs in pancreatic cancer

LncRNAs are not translated into proteins but can modulate physiological processes such as growth, metabolism, and aging. They exert epigenetic modifications and regulate gene expression during and after transcription.^126^ Several lncRNAs were shown to be correlated with the prognosis of pancreatic cancer. Aberrant expression of these lncRNAs was shown to be involved in pancreatic cancer cells proliferation, invasion, and metastasis. Additionally, they interact with intracellular signaling pathways and modulate the expression of several proto-oncogenes and tumor-suppressor genes.^127–129^ A great number of these lncRNAs act through c-Myc. They can promote or inhibit the transcription of c-Myc, hence regulating its oncogenic effects. For instance, it was shown that LINC00261, a methylation-mediated lncRNA, is downregulated in pancreatic cancer. Increased methylation of the promoter of LINC00261 in pancreatic cancer decreases its transcription and is a sign of poor prognosis in pancreatic cancer. LINC00261 decreases c-Myc transcription by methylation of its promoter and inhibits its biologic function.^94,127^ LINC00346 is another lncRNA that promotes tumorigenesis, pancreatic cancer cells proliferation, migration, and invasion and is negatively associated with overall survival (OS) and disease-free survival in pancreatic cancer. It was revealed that LINC00346 is a positive regulator of c-Myc. Indeed, LINC00346 binds to CCCTC-binding factor (CTCF), a transcriptional repressor of c-Myc, to increase the transcription of c-Myc.^130^ LncRNA XLOC_006390 can similarly enhance the biological function of c-Myc by protecting it against ubiquitination and subsequent degradation.^131^ LncRNA HULC is overexpressed in pancreatic cancer which enhances pancreatic cancer cells proliferation and invasion and augments c-Myc expression.^132^ Furthermore, P53 can increase the expression of lncRNA Pvt1 to suppress the transcription of c-Myc and prevent tumor growth.^133^

The relationship between c-Myc and non-coding RNAs seems to be mutual and c-Myc can also regulate the expression of several non-coding RNAs involved in the pathogenesis of pancreatic cancer.^128^ For instance, CCAT1 is a lncRNA that is highly expressed in pancreatic cancer and promotes pancreatic cancer cells proliferation and migration. CCAT1 silencing has been associated with pancreatic cancer cell cycle arrest and decreased cyclin D1 expression. It was revealed that c-Myc increases the expression of CCAT1 by targeting its promoter. Consistently, downregulation of c-Myc has been associated with decreased CCAT1 expression.^128^

Interestingly, it was uncovered that dysregulated expression of these lncRNAs is also involved in the chemoresistance of pancreatic cancer cells. For instance, it was shown that overexpression of lncRNA GSTM3TV2 is associated with overexpression of c-Myc in pancreatic cancer cells and gemcitabine resistance.^134^ The interaction between lncRNAs and c-Myc has been implicated in the metastasis and chemoresistance of several cancers.^135^ Besides, it was shown that these non-coding RNAs can interact with c-Myc to regulate cancer cells metabolism.^136^ Herein, it was shown that XLOC_006390, a lncRNA, promotes c-Myc-mediated expression of glutamate dehydrogenase 1 to increase glutamate metabolism in pancreatic cancer.^131^ Regarding the interaction between c-Myc and lncRNAs in pancreatic cancer and the importance of these lncRNAs in pancreatic cancer, pharmacological inhibition of c-Myc can attenuate the deleterious effect of numerous lncRNAs in pancreatic cancer (Table 1). Also, lncRNAs mimics or inhibitors can be used to regulate the function of c-Myc. This will improve chemoresistance and inhibit tumor cells proliferation, invasion, and metastasis.

**Table 1.** Interaction of different lncRNAs and microRNAs with c-Myc in pancreatic cancer

Author	None-coding RNA	Micro-/lnc-RNA	Finding
*Liu et al. Zhai et al.*	LINC00261	*lncRNA*	LINC00261 decreases c-Myc transcription via methylation of its promoter.
*Peng et al.*	LINC00346	*lncRNA*	LINC00346 binds to CCCTC-binding factor (CTCF) to attenuate its inhibitory effect on c-Myc expression.
*He et al.*	XLOC_006390	*lncRNA*	XLOC_006390 stabilizes c-Myc by preventing its ubiquitination and subsequent degradation.
*Ou et al.*	HULC	*lncRNA*	HULC promotes c-Myc expression to increase pancreatic cancer cells proliferation.
*Yu et al.*	CCAT1	*lncRNA*	C-Myc increases the transcription of CCAT1 in pancreatic cancer. Subsequently, CCAT1 enhances the expression of cyclin D1 to increase pancreatic cancer proliferation.
*Xiong et al.*	GSTM3TV2	*lncRNA*	GSTM3TV2 promotes the expression of c-Myc in pancreatic cancer and is associated with chemoresistance.
*Olivero et al.*	*Pvt1*	*lncRNA*	*P53 promotes the expression of Pvt1. Subsequently, Pvt1 suppresses the expression of c-Myc to prevent tumor growth.*
*He et al.*	XLOC_006390	*lncRNA*	XLOC_006390 increases glutamate bioavailability in cancer cells through upregulation of c-Myc.
*Dey et al.*	miR-29a	*microRNA*	*C-Myc decreases the expression of* miR-29a to prevent its inhibitory effect on the expression of several oncogenes such as LOXL2, MYBL2 and HGK.
*Azmi et al.*	MiR-145	*microRNA*	MiR-145 downregulates the expression of several proto-oncogenes such as EGFR, MMP1 and c-Myc.
*Liu et al.*	MiR-494	*microRNA*	MiR-494 decreases pancreatic cancer cells proliferation and accelerates their apoptosis by suppressing the expression of c-Myc and SIRT1.
*Sureban et al.*	let-7a	*microRNA*	Let-7a is a tumor suppressor that can downregulate c-Myc expression in pancreatic cancer.

### Interaction between c-Myc and microRNAs in pancreatic cancer

MicroRNAs are non-coding RNAs that can post-transcriptionally regulate genes expression, hence, they can be used to modulate specific genes expression.^137^ Yet several microRNAs were shown to interact with c-Myc. C-Myc represses the expression of miR-29a to attenuate its inhibitory effect on the expression of several oncogenes such as lysyl oxidase-like 2 (LOXL2), MYB proto-oncogene like 2 (MYBL2), hematopoietic progenitor kinase/germinal center kinase-like kinase (HGK), and neuroblastoma RAS viral oncogene homolog (NRAS). These genes particularly LOXL2 are overexpressed in pancreatic cancer and the competent function of miR-29a can downregulate the expression of these oncogenes and decrease pancreatic cancer growth.^138^

MiR-145 is downregulated in pancreatic cancer. It negatively regulates pancreatic cancer cells growth and migration. MiR-145 downregulates the expression of several proto-oncogenes such as epidermal growth factor receptor (EGFR), MMP1, and c-Myc.^139^ Similar to miR-145, miR-494 is downregulated in pancreatic cancer cells. MiR-494 suppresses the expression of c-Myc and SIRT1 to inhibit pancreatic cancer cells proliferation and stimulate their apoptosis.^140^ Moreover, decreased expression of miR-494 has been associated with larger tumor size, lymphatic involvement, distant metastasis, and poor prognosis in patients with pancreatic cancer.^140^ A miR-34a mimic in combination with polo-like kinase 1 (PLK1) oncogene silencing could effectively decrease the expression of c-Myc and decrease pancreatic cancer cells viability.^141^ It was shown that c-Myc promotes the expression of Lin-28B RNA binding proteins to decrease the expression of let-7 family in pancreatic cancer.^142,143^ Interestingly, let-7a can also downregulate c-Myc expression, therefore, their interaction can be mutual.^143–145^

In this section, some interactions between c-Myc and microRNAs were mentioned in pancreatic cancer (Table 1). Although, there are many more found in other types of tumor that can be replicated in pancreatic cancer. Expanding our knowledge surrounding these interactions helps to find new methods of cancer chemotherapy.

### Different strategies to target c-Myc

#### Direct targeting of c-Myc

Han *et al*. developed small molecule c-Myc inhibitors such as MYCi361 and MYCi975 which can directly target c-Myc inside the cells and promote its phosphorylation on threonine-58 and thereby stimulate its ubiquitination and degradation.^116^ MYCi361 (initially at 50 mg/kg bid for 2 days, then 70 mg/kg/day for 9 days or 55 mg/kg/day, 3 consecutive days a week for 2 weeks) and MYCi975 (50 mg/kg bid for 2 or 3 days) suppressed tumor growth in mice and enhanced intra-tumor immune cells infiltration. Moreover, the compound improved tumor cells sensitivity to anti-PD1 immunotherapy.^116^

Because of structural characteristics, c-Myc needs to make a heterodimer with Myc-associated factor X (MAX) and thereby bind to DNA.^146^ KSI-3716 is a c-Myc inhibitor that hinders c-Myc/MAX heterodimer binding to the promoter of target genes. This property could effectively inhibit c-Myc-mediated overexpression of different genes such as cyclin D2, CDK4. Intravesical administration of KSI-3716 at a dose of 5 mg/kg could significantly inhibit bladder tumor growth in murine model.^147^ KSI-1449 and KSI-2302 can similarly inhibit the formation of c-Myc/Max/DNA complex, thereby preventing the biologic activity of c-Myc.^148^

Similar to KSI-3716, sAJM589 (with an IC_50_ of 1.8 ± 0.03 μM) is another small molecule that can dose-dependently hamper c-Myc/MAX heterodimer formation. sAJM589 could effectively decrease the expression of c-Myc target genes and even decrease the bioavailability of c-Myc in Burkitt lymphoma cell model.^149^ C-Myc/MAX interaction is necessary for binding to the E-box of target genes and without this heterodimer, c-Myc remains non-functional.^149^ Mycro3, another inhibitor of c-Myc/MAX dimerization, could significantly promote pancreatic cancer cells apoptosis.^66^ Mycro3 (up to 100 mg/kg/day for 2 weeks) also lead to a drastic decrease in tumor cells proliferation and shrinkage in tumor size.^66^

Another inhibitor of c-Myc/MAX dimerization, 10,058-F4, could increase apoptosis in pre-B cell ALL cell line and improve cancer cells sensitivity to other drugs such as dexamethasone and vincristine.^150,151^ The same compound could also improve the sensitivity of acute promyelocytic leukemia (APL) to arsenic trioxide.^152^ Treatment with 10,058-F4 also reduced pancreatic cancer cells viability by accelerating their apoptosis. It partly inhibited glycolysis and led to pancreatic cancer cell cycle arrest at G1/S transition. Also, treatment with 10,058-F4 (with an IC_50_ of 17 and 25 μM in different cell cultures) markedly improved the effect of gemcitabine on pancreatic cancer.^153^

Taken together, there are three mechanisms to directly target c-Myc: 1- Targeting c-Myc itself and accelerating its degradation. 2- Prevention of c-Myc/MAX heterodimerization. 3- Prevention of c-Myc/MAX/DNA complex formation.

#### Indirect targeting of c-Myc

Direct targeting of c-Myc with a potent inhibitor seems to be difficult because of structural complexity and nuclear localization.^154^ C-Myc lacks a fixed structure and has no binding pocket for drugs.^155^ Also, c-Myc possesses a wide range of interactions with different components of intracellular signaling pathways, transcriptional and translational complexes, and non-coding RNAs. Indirect inhibition of c-Myc can be achieved by epigenetic silencing of its gene, transcriptional, post-transcriptional, and translational alterations during its expression. Even, different mechanisms have been proposed to indirectly weaken c-Myc stabilization or prevent its effect on target genes.

#### Bromodomain and extraterminal (BET) inhibitors

BET proteins such as BRD2, BRD3, and BRD4, are nuclear proteins that regulate protein-protein interaction and modify gene transcription by exerting epigenetic effects after binding to acetylated lysine residues on histones and main transcriptional factors.^156^ BET proteins such as BRD2 and BRD3 upregulate c-Myc expression and BET inhibitors such as JQ1 and OTX015 vigorously downregulate the expression of c-Myc.^157,158^ BET inhibitors have been proposed as a new therapeutic option for c-Myc-dependent tumors and have shown promising results in primary studies.^159^ BET inhibitors showed satisfying efficacy in the treatment of pancreatic cancer in cell culture and mice model of pancreatic cancer.^117,160,161^ JQ1 (50 mg/kg intraperitoneal twice weekly), a BET inhibitor, markedly decreased tumor volume in mice with pancreatic cancer transplant.^160^ Their effect on pancreatic cancer is associated with the downregulation of c-Myc. Consistently, c-Myc upregulation reverts the therapeutic effect of BET inhibitors on pancreatic cancer and is responsible for resistance to BET inhibitors.^160–162^ BET inhibitors such as I-BET762 could lead to pancreatic cancer cell cycle arrest in G0/G1 phase and inhibit the proliferation and metastasis of pancreatic cancer cells. Besides, I-BET762 could promote the sensitivity of pancreatic cancer cells to gemcitabine.^163^ I-BET762 (30 mg/kg/day for 13 days) also decreased tumor size in mice with pancreatic cancer xenograft and markedly enhanced the effect of gemcitabine on pancreatic cancer xenograft.^163^ BET inhibitors can suppress several oncogenic pathways other than c-Myc-dependent pathways which can increase their efficacy as chemotherapeutic agents.^164^

#### PP2A

PP2A, a serine/threonine phosphatase, is a tumor suppressor which can dephosphorylate c-Myc and decrease its stabilization. The regulatory subunit of PP2A, B56α, plays a critical role in PP2A-mediated c-Myc destabilization and its following ubiquitination and degradation. Consistently, B56α knockdown is associated with c-Myc overexpression and overactivity.^165–167^ Interestingly, Farrington *et al*. revealed that the use of small-molecule activators of PP2A (SMAPs) in different cancers such as Burkitt lymphoma, KRAS-driven non–small cell lung cancer, and triple-negative breast cancer has been associated with decreased c-Myc bioavailability and diminished tumor cells proliferation.^168^ Similarly, B56α subunit knockdown has been associated with deregulated c-Myc activity and spontaneous tumor development in the liver of mice.^169^ Endogenous inhibitors of PP2A and cancerous inhibitors of PP2A are markedly overexpressed in pancreatic cancer and enhance the expression and stabilization of c-Myc in human pancreatic cancer.^167^ Also, inhibition or knockdown of these inhibitors of PP2A attenuated the tumorigenic potential of pancreatic cancer cells and hindered tumor growth in cell culture and animal model.^167^ Additionally, pharmacological activation of PP2A with FTY-720 (3 mg/kg/day intraperitoneal) notably decreased c-Myc protein levels and significantly decreased pancreatic cancer growth in both cell culture and mice model.^170^ Hence, SMAPs can be alternatively used to indirectly inhibit c-Myc in pancreatic cancer which resulted in acceptable efficacy in vitro and in vivo.^74^

#### Targeting c-Myc by inhibiting its upstream signaling pathways

As noted, c-Myc is downstream of three major proliferative pathways. Blocking each one of these pathways can partly downregulate c-Myc expression. For instance, ERK2 inhibition in lung cancer cells could decrease c-Myc expression. Attenuation of ERK/c-Myc axis in lung cancer resulted in reduced expression of proteins involved in G1-S progression and led to G1 phase arrest.^171^ In particular, targeting PI3K/AKT/mTOR pathway seems to be more effective. mTOR controls different molecules such as 4EBP1 and S6 which are crucial for the initiation and elongation phase of ribosomal translation of c-Myc mRNA.^172^ Furthermore, mTOR regulates the translation of translational factors themselves and many other proteins that their presence is indispensable for tumor cells proliferation.^172^ PI3K inhibitors, AKT inhibitors, mTOR inhibitors, and combinatorial PI3K/mTOR inhibitors have developed and are passing their clinical trials.^173^ Perhaps, use of these compounds can improve the management of pancreatic cancer.

#### Histone deacetylase (HDAC) inhibitors

By deacetylating the chromatin, HDACs can reprogram the expression of several proto-oncogenes and tumor-suppressor genes.^174^ Dysregulation of HDACs has been implicated in the pathogenesis of pancreatic cancer.^175^ HDAC inhibitors showed promising results in pancreatic cancer and could markedly inhibit tumor growth, prevent tumor cells migration and improve chemoresistance.^175,176^ HDAC inhibitors can promote the expression of tumor suppressor genes, hence, repressing tumor growth.^177^ It was unveiled that HDAC1 inhibition vigorously attenuates c-Myc-induced tumorigenesis.^177^ C-Myc and HDAC both are involved in the regulation of gene transcription and proliferation. Likewise, it was uncovered that c-Myc enhances the expression of HDAC2 in pancreatic cancer and thereby suppresses the expression of cyclin G2 (CCNG2), an inhibitor of tumor growth.^178^ Zhu *et al*. unveiled that HDAC7 can increase the transcription of c-Myc to increase the proliferation of colon cancer cells.^179^ Likewise, HDAC6 inhibition led to downregulation of c-Myc and hindered pancreatic cancer cells proliferation.^180^ Also, HDAC inhibition could strongly decrease c-Myc expression in lymphoma cells and non-small cell lung carcinoma.^181,182^ Liu *et al*. revealed that c-Myc can increase the expression of SIRT2, histone deacetylase class III, in pancreatic cancer, subsequently, SIRT2 reinforces c-Myc expression and enhances pancreatic cancer cells proliferation.^183^ HDACs and c-Myc have a mutual relationship and promote the expression of each other, hence, pharmacological inhibition of one of them can also partly attenuate the biologic function of the other one.

#### Nuclear factor activated T cells (NFAT) inhibition

NFATs are a group of calcineurin-responsive transcription factors that promote the transcription of c-Myc, thereby promoting cancer cells proliferation. NFATs particularly NFAT1c is excessively expressed in pancreatic cancer. Inhibition of calcineurin signaling or knockdown of NFAT1c led to G1 arrest and could drastically decrease pancreatic cancer cells proliferation.^184,185^ NFAT1c binds to serum responsive element in the proximal promoter and prepares local chromatin structure for Ets-like gene 1 (ELK1) activity. ELK1 vigorously increases the transcription of c-Myc and promotes its expression.^185^ Buchholz *et al*. revealed that NFATc1 is a major inducer of c-Myc expression, thereby increasing pancreatic cancer cells proliferation.^184^ Induction of NFAT/c-Myc pathway promoted pancreatic cancer growth and NFAT depletion reduced c-Myc expression and decreased pancreatic cancer growth in vitro and in vivo.^184,185^ Inhibition of NFAT can be a promising method to suppress the transcription of c-Myc and decrease its oncogenic effects.

#### GSKβ inhibition

KRAS promotes the gene expression of GSKβ in pancreatic cancer. GSKβ is heavily involved in cancer cells proliferation, survival, and chemoresistance. Additionally, inhibition of GSKβ was shown to be an effective mechanism to treat pancreatic cancer.^186^ Also, PI3K can upregulate c-Myc through a GSKβ-dependent manner. PI3K/GSKβ/c-Myc is crucial for pancreatic cancer cell cycle progression.^187^ GSKβ inhibition decreases the expression of NF-κB and NF-κB -mediated expression of c-Myc in pancreatic cancer, thereby reducing tumor growth.^188^

#### Insulin-like growth factor-2 mRNA-binding protein 1 (IGF2BP1 or IMP1) inhibitor

IMP1 is overexpressed in pancreatic cancer and higher expression of IMP1 predicts a poor prognosis of pancreatic cancer. Decreased expression of miR-494 leads to overexpression of IMP1 in pancreatic cancer.^189^ Meanwhile, downregulation of IMP1 could decrease pancreatic cancer cell growth in cell culture and mice model.^189^ IMP1 binds to mRNA of oncogenes such as c-Myc which stabilizes them. BTYNB, a potent and selective inhibitor of IMP1, significantly decreased the expression of c-Myc and suppressed the proliferation of IMP1-containing ovarian cancer and melanoma cells.^190^ IMP1 protects the coding region instability sequence of c-Myc mRNA against endonucleolytic cleavage.^191^ Similarly, IMP1 can bind to KRAS mRNA and regulate its expression. Deletion of IMP1 significantly reduced KRAS expression.^192^ Consistently, Zhai *et al*. uncovered that IMP1 binds to and stabilizes c-Myc mRNA in pancreatic cancer, thereby augmenting cancer cells proliferation.^94^ In addition, impaired function of IMP1 was associated with decreased c-Myc expression and reduced tumor cells proliferation in pancreatic cancer cells.^94^ Regarding the effect of IMP1 on the stabilization of c-Myc and KRAS mRNA, IMP1 inhibitors are ideal choices to indirectly target both c-Myc and KRAS in pancreatic cancer.

#### Stabilization of MAX/MAX homodimerization

Previously it was discussed how c-Myc-MAX heterodimerization is involved in binding to E-box of the promoter of target genes. Further, it was mentioned that disruption of c-Myc-MAX interaction can inhibit the oncogenic effects of c-Myc. In this regard, Struntz *et al*. used KI-MS2-008, an asymmetric polycyclic lactam that can stabilize MAX homodimer, thereby preventing its heterodimerization with c-Myc. This new mechanism could markedly downregulate c-Myc-driven transcription and reduce tumor size in mice-bearing hepatocellular carcinoma (0.24 mg/kg/day intraperitoneal) or T cell All (0.06 mg/kg/day intravenous).^193^

#### Oxidized low-density lipoprotein receptor 1 (OLR1) inhibition

OLR1 stimulation increases the expression of c-Myc. Consistently, OLR1 activation plays an important role in the pathogenesis of different cancers particularly pancreatic cancer.^194,195^ OLR1, through upregulation of c-Myc, can promote pancreatic cancer cells proliferation, metastasis, and chemoresistance to gemcitabine. OLR1 is overexpressed in pancreatic cancer. OLR1, through c-Myc, promotes the expression of high mobility group A2 (HMGA2). Similarly, overactivity of OLR1/c-Myc/ HMGA2 axis is associated with poor prognosis among patients with pancreatic cancer.^134,195,196^ HMGA2 is a transcription factor that binds to AT-rich sequences to change the structure of chromatin. It has been implicated in tumorigenesis, EMT, and chemoresistance.^197^ As mentioned, HMGA2 is markedly expressed in pancreatic cancer and higher expression of HMGA2 is associated with lymph node metastasis in pancreatic cancer.^198^ Regarding these findings, pharmacological attenuation of OLR1 can indirectly inhibit the deleterious effects of c-Myc on pancreatic cancer.

#### LncRNAs and microRNAs mimics and inhibitors

It has been previously discussed how different lncRNAs and microRNAs can modulate c-Myc expression possibly through epigenetic, transcriptional, and post-transcriptional modifications. Reinforcement of lncRNAs and microRNAs that can negatively regulate c-Myc expression such as LINC00261, Pvt1, MiR-145, MiR-494, and Let-7a seems to be a new and effective method to inhibit tumor cells proliferation and prevent cancer progression. Besides, inhibition of lncRNAs and microRNAs those can enhance c-Myc expression such as LINC00346, XLOC_006390, HULC, GSTM3TV2, and XLOC_006390 can be useful in the same way. However, non-coding RNAs have been the main focus of a notable proportion of recent investigations, their clinical application still did not receive much attention. They can target c-Myc expression in different stages of gene expression and help to overcome drug-resistant tumors. Interestingly, non-coding RNAs can indirectly affect c-Myc by regulating PP2A and other molecules which are in close relationship with c-Myc.^199^ Even, microRNA-22 can disrupt c-Myc/E-box interaction to attenuate the effect of c-Myc on target genes transcription.^12^ However, some c-Myc-interacting lncRNAs and microRNAs were found by previous studies, a greater number remain to be discovered by future studies.^200^ Their mimics or inhibitors are passing their preclinical studies and will constitute a major part of future clinical practice.^137^

### Targeted therapy for c-Myc: when, how, and where?

C-Myc is normally expressed in non-cancerous tissues and is involved in several physiological processes such as metabolism, growth, apoptosis, and differentiation.^201,202^ C-Myc inhibition may exert serious adverse effects on normal tissues as it was shown that c-Myc null mutation was lethal in homozygote mice and decreased fertility in female heterozygote mice.^203^ Although, systemic inhibition of c-Myc in RAS-induced lung adenocarcinoma in mice showed that the effect of c-Myc inhibitors on regenerative tissue is tolerable and completely reversible.^204^ Various mutations that directly affect c-Myc gene or indirectly change its expression by epigenetic modulation can lead to dysregulation and overexpression of c-Myc, impair normal cells growth and contribute to neoplastic transformation.^201,202^ However, c-Myc is a transcription factor whose function is exerted mainly in the nucleus, it has been found that cytoplasmic protein levels of c-Myc independently and negatively correlate with patients survival in pancreatic cancer.^205^ As mentioned throughout the text, c-Myc inhibition showed benefit in different stages of pancreatic cancer as it is involved in pancreatic cancer growth, metabolism, angiogenesis, metastasis, and immune evasion. Therefore, inhibition of c-Myc can be beneficial in different stages of pancreatic cancer. Furthermore, it has been shown that c-Myc overexpression is significantly and positively correlated with histological tumor grade in pancreatic cancer, hence, c-Myc inhibition may provide greater benefit in advanced pancreatic cancer.^13^

To confine the adverse effects of c-Myc inhibitors can be delivered by nanoparticle systems which allow the clinicians to administer the lower doses of drugs and deliver them to the desired site of action.^206^ This method of drug delivery can change the pharmacokinetics of drugs, prolong their duration of action, and remove the unwanted effects of c-Myc inhibition in normal tissues.^206^ Furthermore, as c-Myc interacts with several signaling pathways and is involved in different cellular processes, simultaneous administration of c-Myc inhibitors and immune-checkpoint inhibitors or other chemotherapeutic agents can exert a synergistic effect on pancreatic cancer and contribute to overcoming chemoresistance.^117,153^ Therefore, c-Myc inhibitors in combination with other chemotherapeutic agents and in form of nanoparticulated drugs can provide the utmost benefits particularly for those having higher levels of c-Myc expression in their cancer cells.

## Conclusion and future direction

C-Myc is a transcription for numerous oncogenes. In addition, the effect of several oncoproteins converges on c-Myc and they exert their oncogenic effects by upregulating c-Myc. Hence, inhibition of c-Myc can disrupt a series of oncogenic transformations. C-Myc overexpression enhances proliferation, invasion, metastasis, angiogenesis and immune evasion in pancreatic cancer and c-Myc inhibition attenuates chemoresistance and enhances immunotherapy. During recent years several methods developed to target the “undruggable” c-Myc. Yet, there is not any clinical trial attempting to measure the effect of c-Myc in pancreatic cancer. Regarding the poor response of pancreatic cancer to current chemotherapy protocols, the prevalence of c-Myc deregulation in pancreatic cancer, and the promising results of preclinical studies, it is worth investigating the effect of different inhibitors of c-Myc in clinical trials of pancreatic cancer.

## Data Availability

Data sharing is not applicable to this article as no new data were created or analyzed in this study.
